# Nicotinamide Riboside Vitamin B3 Mitigated C26 Adenocarcinoma–Induced Cancer Cachexia

**DOI:** 10.3389/fphar.2021.665493

**Published:** 2021-06-28

**Authors:** Jong Min Park, Young Min Han, Ho Jae Lee, Yong Jin Park, Ki Baik Hahm

**Affiliations:** ^1^ College of Oriental Medicine, Daejeon University, Daejeon, South Korea; ^2^ Seoul Center, Korea Basic Science Institute, Seoul, South Korea; ^3^ Lee Gil Ya Cancer and Diabetes Institute, University of Gachon, Incheon, South Korea; ^4^ GI Medics, Seoul, South Korea; ^5^ CHA Cancer Preventive Research Center, CHA Bio Complex, Pangyo, South Korea; ^6^ Medpacto Research Institute, Medpacto, Seoul, South Korea

**Keywords:** nicotinamide ribose, cancer cachexia, NAMPT1, sarcopenia, muscle atrophy, inflammation

## Abstract

Nicotinamide riboside (NR), vitamin B3, is a substrate for nicotinamide adenine dinucleotide (NAD^+^)–consuming enzymes and is a coenzyme for hydride-transfer enzymes, including adenosine diphosphate (ADP)–ribose transferases, poly (ADP-ribose) polymerases, cADP-ribose synthases, and sirtuins, which play a central role in the aging process, neurodegenerative processes, and myopathy. Since cancer cachexia is a disease condition presenting with weight loss, skeletal muscle atrophy, and loss of adipose tissue in patients with advanced cancer, we hypothesized that NR intake could ameliorate sarcopenia. In this study, we investigated whether preemptive administration of NR ameliorated C26 adenocarcinoma–induced cancer cachexia and explored anti-cachexic mechanisms focused on the changes in muscle atrophy, cachexic inflammation, and catabolic catastrophe. Dietary intake of the NR-containing pellet diet significantly attenuated cancer cachexia in a mouse model. Starting with significant inhibition of cachexic factors, tumor necrosis factor alpha, and interleukin-6, NR significantly inhibited muscle-specific ubiquitin-proteasome ligases, such as atrogin-1, muscle RING-finger protein-1 (MuRF-1), mitofusin-2, and peroxisome proliferator–activated receptor gamma coactivator-1-alpha (PCG-1α). Significant inhibition of epididymal fat lipolysis was noted with significant inhibition of adipose triglyceride lipase (ATGL) gene. Furthermore, NR administration significantly increased the levels of crucial enzymes involved in the biosynthesis of NAD^+^ and nicotinamide phosphoribosyl transferase and significantly inhibited the NAD^+^-sensitive deacetylase sirtuin 1 (SIRT1). Preemptive intake of NR in patients vulnerable to cachexia can be a preemptive option to ameliorate cancer cachexia.

## Introduction

Cancer cachexia is characterized by a significant reduction in body weight, resulting predominantly from loss of skeletal muscle and adipose tissue in patients with advanced cancer. Since complete treatment of cancer as well as the associated and complicated cachexia, except for the early detection of cancer, is not easy, and recovery from the underlying condition causing cancer is impregnable, troublesome cancer cachexia is an unmet medical need. Therefore, as a scientific approach to cancer cachexia, many attempts have been made to inhibit cancer cachexia by targeting the inflammatory cytokines, tumor necrosis factor alpha (TNF-α) and interleukin (IL)-6. Downstream of these cytokine-associated cachexic factors, muscle atrophy–associated genes, such as *muscle RING-finger protein-1* (*MuRF-1*) and *atrogin-1*, resulting from the induction of the ubiquitin-proteasome system (UPS) *E3 ligase* genes that mediate the degradation of myofibrillar proteins through the ubiquitin-proteasome pathway, lead to multiple catabolic catastrophes and weaknesses; several approaches are under investigation, for instance, exercise, nutritional intervention, appetite improvement, and some phytochemicals; however, a solution is not available.

The role of nicotinamide adenine dinucleotide (NAD^+^) metabolism in health and disease is of increased interest because nicotinamide can protect tissues, and NAD^+^ metabolism has been implicated in a variety of disease states in addition to extending the lifespan (Belenky et al., 2007; Sauve, 2008). As a result, enzymes including poly (ADP-ribose) polymerases (PARP), mono-ADP-ribosyltransferases, and *sirtuin* and/or NAD^+^ metabolism could be targeted for the therapeutic benefit of patients with cachexia and sarcopenia because the NAD^+^ precursor, NR, has been implicated in improving myopathy and muscle atrophy (Bogan and Brenner, 2008).

In this study, under the hypothesis that NR supplementation can ameliorate sarcopenia in relation to cancer cachexia, we administered NR-containing pellet diets preemptively in C26 adenocarcinoma–induced cancer cachexia mice models and found that dietary administration of NR can be a preemptive treatment in patients who are at high risk for cancer cachexia.

## Materials and Methods

### Cell Cultures

Mouse colon carcinoma cells, Colon26 (C26) cells, were obtained from Cell Lines Service GmbH (CLS, Eppelheim, Baden-Württemberg, Germany) and maintained according to the CLS’s instructions. C26 cells were maintained in RPMI-1640 medium containing 10% fetal bovine serum (FBS, Sigma-Aldrich, St Louis, MO, United States) and 1% antibiotic anti-mycotic solution (Sigma-Aldrich, St Louis, MO, United States) at 37°C in a humidified atmosphere composed 5% CO_2_ incubator.

### Nicotinamide Riboside–Containing Pellet Diets

We administered a mouse diet supplemented with NR at 200 or 400 mg/kg daily for 3 weeks as pellet diet.

### Animal Experimental Procedure for Cancer Cachexia

Six-week-old male *Balb/c* mice (total *n* = 40, Orient Animal, Seoul, Korea) were randomly divided into four groups: normal group (*n* = 10), cachectic C26 adenocarcinoma–bearing group (*n* = 10), cachectic C26 adenocarcinoma–bearing group with 200 mg/kg containing pellet diets (*n* = 10), and cachectic C26 adenocarcinoma–bearing group with 400 mg/kg containing pellet diets (*n* = 10). Cachectic C26 adenocarcinoma–bearing groups were shaved on the legs side and injected in their right flank with 1 × 10^7^ C26 cells. According to the method of Acharyya et al. (2004) and Murphy et al. (2013)with minor modifications*,* cells were counted using a hemocytometer, suspended in 100 μl of sterilized phosphate-buffered saline and then C26 cells were injected into the mice. For 3 weeks, we fed a normal pellet diet or pellet diet with NR with daily measurement of intake. We restricted feeds to 100 g per group to measure reduced food intake due to anorexia. Following this experimental regimen, mice were monitored every 3 days, including body weight and food intake. Leg muscles were isolated and subjected to further histologic examination, enzyme-linked immunosorbent assay (ELISA), Western blotting, and reverse transcription polymerase chain reaction (RT-PCR). Animal studies were carried out in accordance with protocols approved by the Institutional Animal Care and Use Committee of CHA University CHA Bio Complex after IRB approval (IACAC 2019-0601).

### Enzyme-Linked Immunosorbent Assay

On day 21 of the experiments, the mice were sacrificed. After the sacrifice of the animals, blood was collected for ELISA assay. After centrifugation at 3,000 rpm, 4°C for 30 min, the IL-6 and TNF-α levels in the supernatant were measured by ELISA, and concentration of IL-6/TNF-α is expressed as pg/mg protein. The process was performed strictly as prescribed in IL-6 or TNF-α ELISA kit manuscript (R&D SYSTEM, Minneapolis, MN, United States). All samples were measured at individual levels, and each sample was analyzed in triplicate manner, taking a mean of the three determinations.

### Reverse Transcription-PCR

Total RNA was isolated from leg muscle tissues using TRIzol reagent (Life Technologies, Milan, Italy), and 1–5 mg of each total RNA was transcribed to cDNA using cDNA synthesis kit (TOYOBO, Osaka, Japan). The PCR mixture contained 2× PCR MasterMix (Bioneer, Daejeon, South Korea), autoclaved water, primer (10 pmole/lL), and cDNA in final volume of 20 μl. PCR was performed over 30 cycles of 94°C for 30 s, 52°C for 30 s, and 72°C for 30 s. Oligonucleotide primers were purchased from Bioneer (Daejeon, South Korea). Oligonucleotide primers for IL-6, Atrogin-1, MuRF-1, Mfn-2, and GAPDH are shown in Table 1. Each PCR product was directly loaded onto 1.5% agarose gels and stained with Redsafe (iNtRON Biotechnology, Cheonan, South Korea).

**TABLE 1:** Primer sequences in this experiment.

Primer	Forward	Reverse
IL-6	GGGACTGATGCTGGTGACAA	TAACGCACTAGGTTTGCCGA
Mfn-2	GCT​CGG​AGG​CAC​ATG​AAA​GT	ATC​ACG​GTG​CTC​TTC​CCA​TT
Atrogin-1	CTGAATAGCATCCAGATCAGCAGG	TTGATAAAGTCTTGAGGGGAAAGTG
MuRF-1	AAATGCTATGGAGAACCTGGA	GTCCTTGGAAGATGCTTTGTAA
GAPDH	AATGTATCCGTTGTGGATCT	TCCACCACCCTGTTGCTGTA

### Western Blot Analysis and Antibodies

Extracted tissues were washed twice with PBS and then lysed in ice-cold cell lysis buffer (Cell Signaling Technology, Denver, MA, United States) containing 1 mM phenylmethylsulfonyl fluoride (PMSF, Sigma-Aldrich, St Louis, MO, United States). After 20 min of incubation, samples were centrifuged at 10,000 × g for 10 min. Supernatants were then collected. Proteins in lysates were separated by sodium dodecyl sulfate polyacrylamide gel electrophoresis (SDS-PAGE) and transferred to polyvinylidene fluoride (PVDF) membranes, which were incubated with primary antibodies, washed, incubated with peroxidase-conjugated secondary antibodies, rewashed, and then visualized using an enhanced chemiluminescence (ECL) system (GE Healthcare, Buckinghamshire, United Kingdom) and the relative amounts of proteins associated with specific antibody were quantified using Lumi Vision Imager software (TAITEC). Primary antibodies for Western blotting against NAMPT1, SIRT1, Pax7, ubiquitin, p-p38, ATGL (adipose triglyceride lipase), PARP-1, caspase-8, Bax, and Cyt c were from Cell Signaling Technology (Danvers, MA, United States). Antibodies against MuRF-1 (muscle ring-finger protein-1), Mfn-2 (mitofusin-2), p-AMPKα 1/2, PGC-1α (peroxisome proliferator–activated receptor gamma coactivator-1-alpha), p-JNK, p-ERK, and β-actin were from Santa Cruz Biotechnology (Dallas, TX, United States) Antibody against Atrogin-1 was from ECM biosciences (Versailles, KY, United States). Anti-mouse and anti-rabbit horseradish peroxidase–conjugated secondary antibodies were from Cell Signaling (Danvers, MA, United States).

### Statistics

Statistical analysis results are expressed as the mean standard deviation (SD). Statistical analyses were conducted with GraphPad Prism (GraphPad Software, La Jolla, CA, United States) and SPSS software (version 12.0; SPSS Inc., Chicago, IL, United States). Statistical significance between groups was determined by a multivariate test, Kruskal–Wallis test. Statistical significance was accepted at *p* < 0.05.

## Results

### Nicotinamide Riboside–Containing Pellet Diet Ameliorated C26 Adenocarcinoma–Induced Cancer Cachexia Irrespective of the Size/Volume of Transplanted Tumors

**FIGURE 1:**
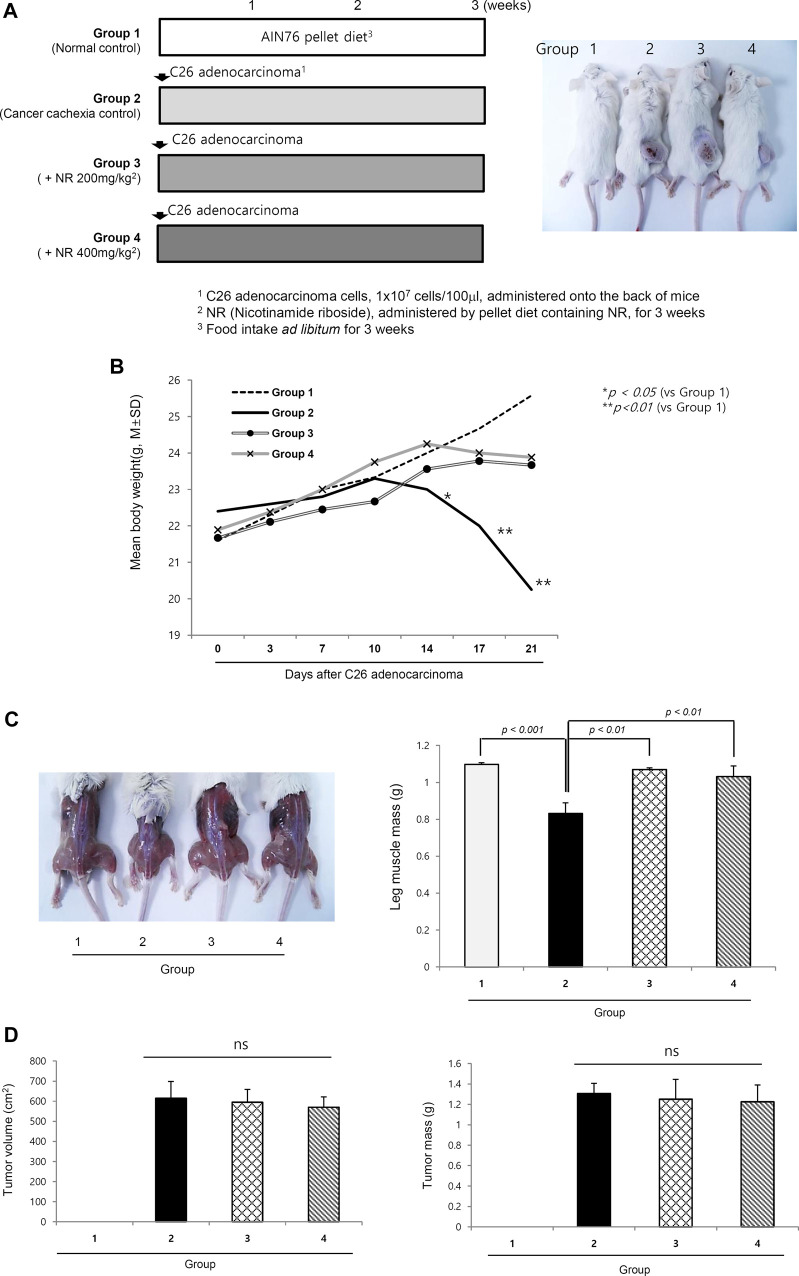
Dietary intake of NR as pellet diet to mitigate C26 adenocarcinoma–induced cancer cachexia. **(A)** Schematic protocol for experiment *Balb/c* mice were administered with 1 × 10^7^ cells/ml on side of abdomen. Right picture shows representational tumors according to the groups. Tumor growth did not differ according to the groups. **(B)** Daily measurement of body weight according to the groups. Body weights after 2 weeks were significantly decreased in Group 2 compared to Group 3 and Group 4. **(C)** Representational gross morphology of thigh including gastrocnemius muscle. Significant atrophy was noted in Group 2 (*p < 0.01*). Using imaging analysis, individual muscle mass of leg was measured and averaged according to the groups. The mean size and volume of resected C26 adenocarcinoma xenograft after 3 weeks. **(D)** Mean tumor volume and tumor masses according to the groups. Though different in body weights and general condition, NR administration did not affect either tumor volume or tumor masses. Though omitted, the mean amounts of daily food intake were not differed according to the groups, suggesting that changes in either appetite or food consumption was not differed according to the groups.

As shown in Figure1A, four groups (*n* = 10/group) were included as follows: normal control, cancer cachexia control, and cancer cachexia administered 200 mg/kg NR and 400 mg/kg NR as pellets in their daily diet. The experiments continued until 3 weeks after C26 cell administration because 20% weight loss was noted in the cachexia control group (25–30% weight loss around 4 weeks led to mortality in some). Although there were no significant differences in the transplanted C26 cells between groups, weight changes were significantly different between the control group and the groups administered the NR-containing pellet diet (*p* < 0.05, Figure 1B). Significant differences in body weight were evident in both leg muscles and general mouse morphology. As seen in Figure 1C, the thigh and gastrocnemius muscles were significantly decreased in the cachexia group 2, whereas they were well-preserved in the NR-administered group, Group 3 and Group 4 (*p* < 0.01, Figure 1C). However, when measuring each tumor growth on the buttocks, no significant difference in either tumor volume or tumor size was observed (Figure 1D), indicating that the NR diet prevented C26 adenocarcinoma–induced muscle atrophy.

### Nicotinamide Riboside Significantly Decreased Cachexic Cytokines, Tumor Necrosis Factor Alpha, and Interleukin-6

**FIGURE 2:**
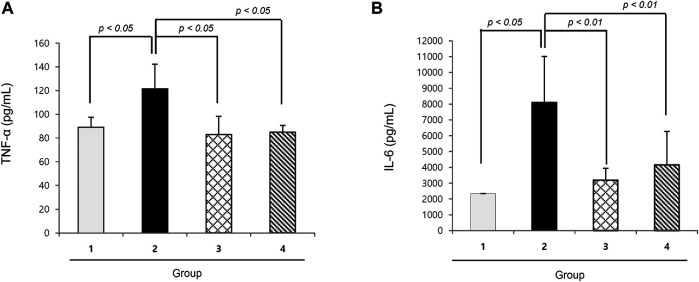
Mean sera levels of cachexic cytokines, TNF-α and IL-6 according to the groups **(A)** TNF-α and **(B)** IL-6. TNF-α and IL-6 were significantly increased in Group 2, cancer cachexia group. Their levels were significantly decreased in the group treated with NR administration.

TNF-α and IL-6 are well-known cytokines that provoke cancer cachexia in the C26 adenocarcinoma model, where we measured the serum levels of both. As seen in Figure 2, the levels of TNF-α and IL-6 were significantly increased in the cachexia control group than in the normal control group (*p* < 0.01). However, groups 3 and 4 that were administered 200 mg or 400 mg/kg NR mixed in pellet diet presented significantly decreased TNF-α and IL-6 levels (*p* < 0.01).

### Changes in Mitogen-Activated Protein Kinase and Muscle-specific Mediators

**FIGURE 3:**
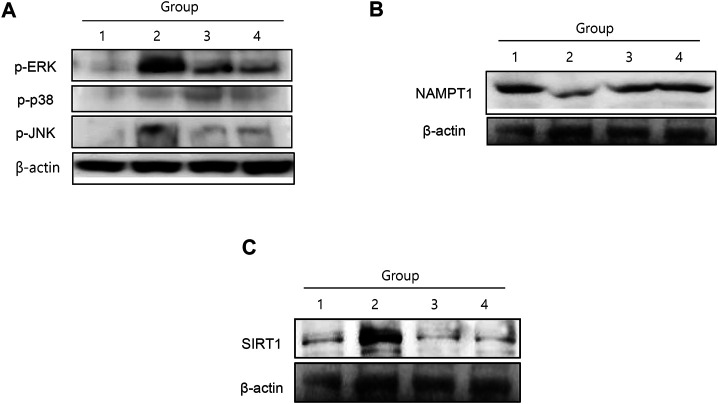
MAPKs, NAMPT, and SIRT1 changes according to the group. **(A)** C26-induced cancer cachexia was associated either ERK and JNK activation, then NR administration significantly inactivated these cachexia-associated MAPKs. **(B)** NAMPT1 was significantly decreased in cancer cachexia group, suggesting incomplete conversion of NAD^+^ in cancer cachexia, whereas NR administration maintained NAMPT1 expression. **(C)** SIRT1 was significantly increased in cancer cachexia group, but not in the NR-treated group, suggesting that NR administration did not affect SIRT1-mediated muscle atrophy.

Among mitogen-activated protein kinases (MAPKs), cancer cachexia was significantly associated with the activation of either extracellular-signal regulated kinase 1/2 (ERK1/2) or c-Jun N-terminal kinase (JNK) (Figure 3A). ERK1/2 and JNK phosphorylation was significantly increased in group 2, whereas both kinases were significantly inactivated by NR administration (Figure 3A). Nicotinamide phosphoribosyl transferase (NAMPT), an enzyme essential for maintaining the NAD^+^ pool utilizing nicotinamide riboside kinase 1 (NRK1) to synthesize NAD^+^ from NAD^+^ precursors (Schondorf et al., 2018), was significantly reduced in group 2, but was restored in the NR group. SIRT1 expression was significantly increased in group 2, but attenuated in the group treated with NR (Figure 3B). In contrast, although SIRT1 has been known to stimulate muscle regeneration, in our investigation, SIRT1 was significantly increased in cancer cachexia, whereas its level was significantly decreased in the group treated with NR (*p* < 0.001, Figure 3C), reflecting the fact that the elevation of SIRT1 might be due to compensatory mechanism against muscle atrophy (Toledo et al., 2011).

### Changes in Muscle-specific Ubiquitin-Proteasome System and Muscle-Related Genes

**FIGURE 4:**
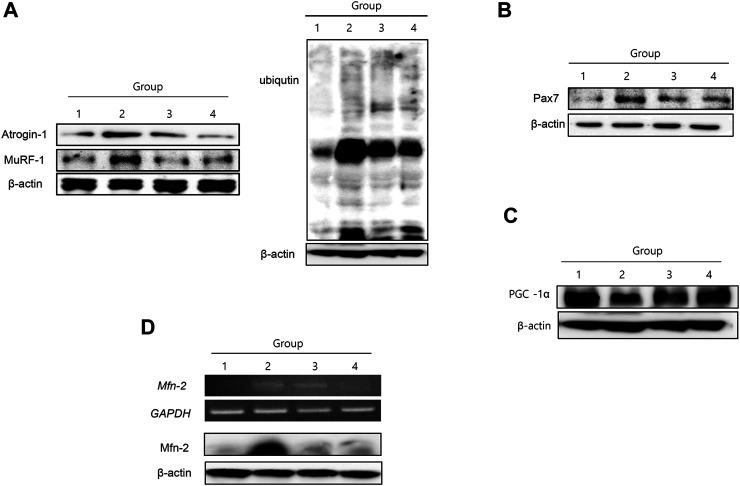
Changes of muscle-specific UPS ligase atrogin-1 and MuRF-1, Pax 7, PCG-1α, and mitofucin-2. **(A)** Western blot for atrogin-1 and MuRF-1 according to the groups **(I)** and UPS ligase according to the groups blotted with ligase **(II)**. **(B)** Western blot for Pax7 and **(C)** Western blot for PCG-1α **(D)** RT-PCR and Western blot for mitofucin-2.

Muscle-specific UPS, *atrogin-1* and *MuRF-1*, were significantly increased in group 2, signifying that muscle-specific ubiquitin-proteasome ligase was enhanced during muscle atrophy in cancer cachexia (Figure 4A). NR treatment significantly decreased *atrogin-1* and *MuRF-1*, resulting in decreased ubiquitin ligase activity in the group treated with NR. Pax7, a transcription factor implicated in muscle atrophy, was significantly increased in group 2 but significantly attenuated in the group treated with NR (Figure 4B). PCG-1α, a marker for muscle regeneration, was significantly decreased in group 2 but was restored in the group treated with NR (Figure 4C). Mitofusin-2, which reflects mitochondrial activity, was significantly increased in group 2, signifying that the degradation of muscle associated with cancer cachexia was significantly increased in group 2, but not in the NR-treated group (Figure 4D).

### Lipolysis and Anti-Proliferation in Cancer Cachexia and Reversal With Nicotinamide Riboside

**FIGURE 5:**
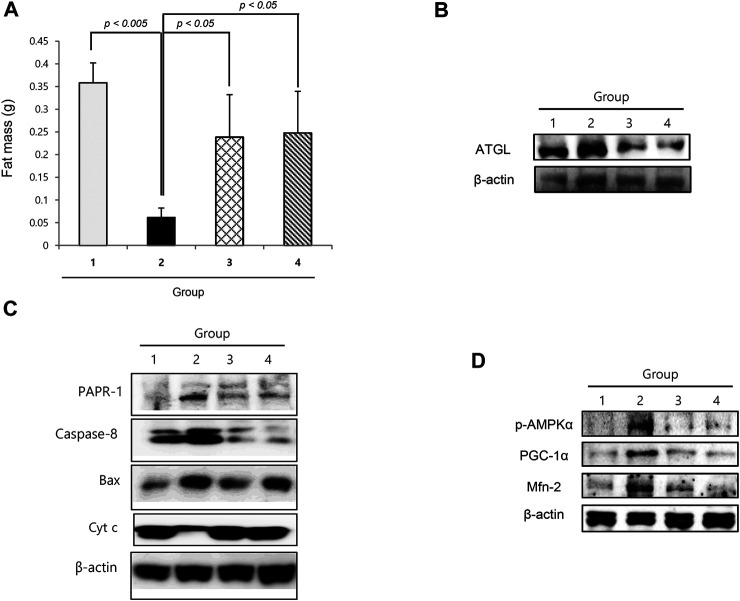
Changes of fat status according to the groups. **(A)** Epididymal fat mass according to the groups. **(B)** Western blot for ATGL. **(C)** Western blot for apoptotic executors according to the groups. **(D)** Western blot for p-AMPKα, PCG-1α, and Mfn-2.

Similar to muscle atrophy, significant fat loss is a remarkable finding in cachexia. As shown in Figure 5A, the fat mass in the epididymal area was significantly decreased in cancer cachexia group 2 (*p* < 0.01), whereas epididymal fat was significantly preserved in the NR group. Adipose triacylglycerol lipase was significantly increased in group 2, leading to significant lipolysis in cancer cachexia, but was significantly decreased in the NR group (Figure 5B). Further exploration of apoptotic executors and substrate revealed the significant increase in caspase-1, Bcl-2–associated X protein (Bax), and PARP-1 in group 2, but a significant decrease in the group treated with NR (Figure 5C).

## Discussion

**FIGURE 6:**
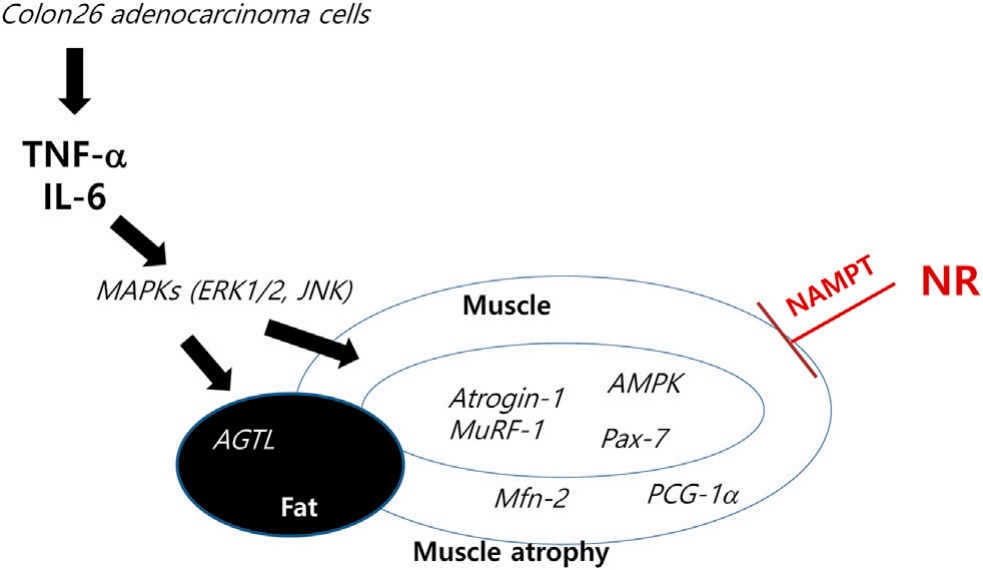
Schematic presentation telling how NR intake tackles the cachexic process. Anti-cachexic effect of preemptive dietary intake of NR vitamin B3 in C26 adenocarcinoma–induced cancer cachexia. Preemptive administration of NR exerted significant protection from muscle atrophy, inflammation, and catabolic disaster featured in cancer cachexia.

About half of all cancer patients exhibit cachexia accompanied with anorexia, loss of body fat, and sarcopenia (Suzuki et al., 2013). Cachexia alone indicates poor quality of life, which could lead to severe weight loss during chemotherapy. Many drugs, including appetite stimulants, such as ghrelin (a 28 amino acid orexigenic gut hormone), mimetics (Khatib et al., 2018; Mohammadi et al., 2019), thalidomide, cytokine inhibitors, such as MABp1 [a natural IgG1k human monoclonal antibody against IL-1α (Prado and Qian, 2019)], steroids, such as progesterone (Gharahdaghi et al., 2019; Solomon et al., 2019), nonsteroidal anti-inflammatory drugs, such as celecoxib (Mantovani et al., 2010; Solheim et al., 2013), and branched-chain amino acids, such as leucine 2–4 g/day or eicosapentaenoic acids (Pappalardo et al., 2015; Prado et al., 2020; Storck et al., 2020), have been investigated in the clinic, whereas others are still under investigation (Ravasco, 2019). Although great progress has been made in understanding the underlying biological mechanisms of cachexia, further development is still awaited. In this miserable state, our current study clearly revealed the efficacious contribution of dietary NR supplementation in a cancer cachexia model in a preemptive manner (Figure 6).

Since NAD^+^ availability may protect skeletal muscle from age-related metabolic decline, dietary supplementation with nicotinamide mononucleotide (NMN) and NR as NAD^+^ precursors appears efficacious in elevating muscle NAD^+^ (Fletcher et al., 2017). Therefore, skeletal muscle cells require NAMPT to maintain NAD^+^ availability. NAMPT catalyzes the conversion of nicotinamide to NMN, which is associated with most of the NAD^+^ formation.

As the pathogenesis of age- or cachexia-associated muscle declines, two key NAD^+^ intermediates, including NR and NMN, have been studied over the past several years (Yoshino et al., 2018) because supplementation using these NAD^+^ intermediates has shown preventive and therapeutic effects against aging and sarcopenia. Although not measured in this investigation, NRK1 is a rate-limiting enzyme for the use of exogenous NR for NAD^+^ synthesis (Ratajczak et al., 2016). Since mammalian cells require the conversion of extracellular NMN to NR for cellular uptake and NAD^+^ synthesis, we measured the changes in SIRT1 instead of NRK because NAD^+^ is a vital redox cofactor and a substrate required for the activity of various enzyme families, including *sirtuin*s and PARP.

Since NR and NAD^+^ also play important roles in the regulation of NAD^+^-consuming enzymes, including *sirtuin*s, PARPs, and CD38/157 ectoenzymes, and NAD^+^ biosynthesis mediated by NAMPT and SIRT1 function together to regulate metabolism, NAD^+^ levels decline during cachexia, causing defects in mitochondrial functions and resulting in sarcopenia (Imai and Guarente, 2014). As seen in our investigation (Figure 3B), NAMPT expression in the cachexia group was significantly increased with NR administration, whereas SIRT1 levels decreased. Restoring NAD^+^ by supplementing NR could dramatically ameliorate cachexia-associated sarcopenia and counteract cancer cachexia (Imai, 2010).

In Figures 3B,C, the contribution of dietary NR administration against cancer cachexia is shown through the changes in NAMPT and SIRT1. Curiously, NR administration decreased SIRT1 expression, which was unexpectedly increased in the cachexia group, a contradictory finding, because of the lack of muscle regeneration during muscle wasting in tumor-bearing animals *via* SIRT1 (Toledo et al., 2011). Pharmacological blockade of NADPH oxidase 4 (nox4), a key regulator of reactive oxygen species in muscle, significantly abrogated cancer cachexia in mice; targeting the SIRT1-nox4 axis seems to be an effective therapeutic intervention for mitigating cancer cachexia (Dasgupta et al., 2020). As an effect of SIRT1, nuclear factor kappa B (NF-κB) signaling is inhibited by deacetylation of the p65 subunit of NF-κB complex and stimulation of oxidative energy production via the activation of AMP-activated protein kinase, peroxisome proliferator–activated receptor-alpha, and peroxisome proliferator–activated receptor gamma coactivator-1-alpha. Therefore, although SIRT1 inhibition disrupts oxidative energy metabolism and stimulates NF-κB-induced inflammatory responses, SIRT1 inhibition is associated with muscle preservation (Kauppinen et al., 2013).

Recently, Hulmi et al. reported about disturbed muscle NAD^+^ homeostasis in experimental cancer cachexia (Hulmi et al., 2020). Muscle proteomics in C26 tumor–bearing (TB) cachectic mice revealed downregulated signatures for mitochondrial oxidative phosphorylation (OXPHOS) and increased acute-phase response (APR). These were accompanied by muscle NAD^+^ deficiency, alterations in NAD^+^ biosynthesis, and decreased muscle protein synthesis. Our findings supported these results such as NAD^+^ depletion, muscle protein degradation, and the elevation of inflammatory cytokine in TB-mice. However, the induction of enzymes of the NAD^+^ biosynthesis pathway such as NAMPT and the reduction of SIRT1, a conserved protein NAD^+^-dependent deacetylase, in TB-mice are inconsistent with our study. Hulmi et al. collected samples 11 days after the C26 cell inoculation, but we collected samples 21 days after the C26 cell inoculation. In our study, the elevation of SIRT1 might be due to compensatory mechanism against muscle atrophy. Further research should also examine by time dependent manner.

The basic action of NR in mitigating cancer cachexia with NR administration, as shown in Figure 2, is significantly decreasing the levels of IL-6 and TNF-α. Different kinds of cancer cells are known to secrete IL-6, a candidate mediator of cachexia, and IL-6 levels correlate with weight loss in some cancer patients (Scott et al., 1996; Moses et al., 2009); moreover, increasing levels of IL-6 in tumor-bearing mice correlated with the development of cachexia (Strassmann et al., 1992). Even better than IL-6, TNF-α is a key inflammatory cytokine in cancer cachexia (Tracey and Cerami, 1994; Lu et al., 2020).

“Preemptive administration” is usually employed for pain control (Szedlak et al., 2018), including the application of multifactorial synergistic medication before pain development. In this condition, the absence of biomarkers that cause cachexia has currently led to a dependence on clinical findings. However, there had been several publications suggesting poor prognosis because of poor muscle mass, weight loss, and poor performance in cancer patients. To date, our study may be the only study showing preemptive efficacy in ameliorating cancer cachexia.

Lipolysis is also a prominent event implicated in cancer cachexia. Inadera et al. (2002) clarified the biological characteristics of a lipid-depleting factor in both 3T3-L1 adipocytes and C26-inoculated mouse cachexia model and found that a reduced quantity of mature sterol regulatory element–binding transcription factor-1, without affecting peroxisome proliferator-activated receptor (PPAR)-γ and CCAAT/enhancer binding protein (C/EBP)-α, resulted in increased lipolysis and reduced lipogenesis. SREBP-1 and C/EBP-alpha are key transcriptional factors involved in lipogenesis. SREBP-1 is a transcription factor that regulates lipid and fatty acid synthesis, energy storage, and acts as intercellular signaling nodes of convergence/divergence. C/EBP-alpha plays a significant role in adipocyte differentiation. For example, the transcription factor C/EBP-alpha is considered to be essential master regulators of adipogenesis. Similar findings were noted in the current study, but dietary NR significantly restored cachexia-induced fat loss under the same molecular changes. In another study by Bing et al. (2006) using a similar cachexia model of MAC16 tumors, adipose tissues from cachexic mice contained shrunken adipocytes and increased fibrosis, with similar findings observed in muscle tissue, causing shrinkage in size, decreased muscle bundles, and some fibrotic changes. Genetic analysis revealed major reductions in mRNA levels of adipogenic transcription factors, such as C/EBP-α and C/EBP-β, PPAR-γ, fatty acid synthase, acetyl-CoA carboxylase, stearoyl-CoA desaturase 1, glycerol-3-phosphate acyltransferase, and sterol regulatory element–binding transcription factor-1.

In conclusion, we found that preemptive dietary intake of NR in preclinical models of cancer cachexia can be a potential regimen to inhibit the occurrence of a catabolic catastrophe, cachexic inflammation, and muscle atrophy. A well-designed randomized clinical trial should be developed to study whether dietary intake of NR can cover the unmet medical needs in cancer cachexia.

In this study, we documented that dietary intake of NR, a vitamin B3, can be a feasible preemptive intervention in patients with advanced cancer who are vulnerable to cachexia. As summarized in Figure 6, C26 adenocarcinoma led to significant cancer cachexia accompanied by muscle degeneration with disturbed muscle regeneration, high TNF-α and IL-6 levels, MAPK activation leading to increased *atrogin-1*/*MuRF-1* levels, and lipolysis. However, preemptive administration of NR led to significant rescue from cancer cachexia. Considering the fact that more than 30% of patients with chronic illness die due to cachexia and more than 50% of patients with cancer because of cachexia, but not due to chemotherapeutic drugs or agents, our investigation revealed that preemptive and dietary intake of NR in patients at high risk of developing cancer cachexia can be a potential regimen to inhibit the occurrence of a catabolic catastrophe, cachexia inflammation, and muscle atrophy. However, well-designed RCTs should be developed for the unmet medical needs in cancer cachexia.

## Data Availability

The raw data supporting the conclusion of this article will be made available by the authors, without undue reservation, to any qualified researcher.
